# Shyness and academic procrastination among Chinese adolescents: a moderated mediation model of self-regulation and self-focused attention

**DOI:** 10.3389/fpsyg.2024.1352342

**Published:** 2024-03-21

**Authors:** Hong Sun, Yang Yu, Chao Peng

**Affiliations:** ^1^College of Teacher Education, Taishan University Shandong Province, Tai’an, China; ^2^Shandong Provincial Qianfoshan Hospital, Jinan, China

**Keywords:** shyness, self-regulation, self-focused attention, academic procrastination, Chinese adolescents

## Abstract

Academic procrastination is a common concern among adolescents, but the correlation between shyness and academic procrastination and the internal mechanisms have not yet been thoroughly investigated. Based on a questionnaire survey with 1,279 Chinese middle school students, this study examined the effect of shyness on academic procrastination and its underlying mechanism of self-regulation and self-focused attention. Results revealed that: (1) shyness significantly predicted academic procrastination. (2) Self-regulation mediated the relationship between shyness and academic procrastination. (3) Self-focused attention played a moderating role in the first half of this mediation process. Specifically, higher level of self-focused attention strengthened the predictive effect of shyness on self-regulation. These results underscored the latent risks and protective factors associated with shyness, self-regulation, and self-focused attention in adolescent academic procrastination. In future research and interventions, attention may be directed towards improving individual internal factors to assist adolescents in effectively addressing issues related to academic procrastination.

## 1 Introduction

Academic procrastination refers to unnecessary and irrational behaviors that lead to the delay of academic tasks (Steel and Klingsieck, 2016). It exerted an adverse impact on students’ academic performance (Cormack et al., 2020) while also influencing their physical and mental health (Flett et al., 2016). Thus, exploring the determinants of academic procrastination becomes crucial in curtailing its prevalence among adolescents and encouraging their healthy development.

The critical determinants influencing academic procrastination were a medley of internal factors such as personality traits, self-regulation; and environmental factors like task complexity or teacher characteristics (Klingsieck, 2013; Steel and Klingsieck, 2016). Notably, the internal factors wield significant swayed over individual behaviors. A deep-dive into these factors can facilitate an enhanced understanding of students varying scholastic performances and diversified learning behaviors, thereby revealing the plethora of causes underpinning student behavior. Hence, an intensive exploration of these internal factors is pivotal to uncovering the mechanisms and repercussions of academic procrastination. This study, embedded within the constructs of Attention Control Theory and Self-focused Attention Theory, aims to unearth the influence and operation of these internal factors—namely shyness, self-regulation, and self-focused attention—on adolescent academic procrastination. The ultimate goal is to panoramically address this issue, thereby furnishing effective recommendations to foster robust academic growth within the adolescent cohort.

### 1.1 Shyness and academic procrastination

Shyness is characterized as the discomfort or inhibition individuals experience in social situations or under social evaluation (Coplan et al., 2004). Both theoretical constructs and empirical evidence suggests a potential association between shyness and academic procrastination.

While direct research establishing a correlation between shyness and academic procrastination was limited, existing studies underscored the significant role of personality traits in fostering academic procrastination (Klingsieck, 2013). Shyness, as a stable personality trait, induced negative emotions that hindered active participation in classroom discussions and discouraged seeking academic assistance (Chen et al., 2021). Furthermore, these emotions extended to impair crucial cognitive processes necessary for initiating individual tasks in academic settings, leading to divided attention and self-doubt (Geng et al., 2021; Leigh et al., 2021), ultimately diminishing task completion proficiency and fostering academic procrastination.

Secondly, Attentional Control Theory (Derakshan and Eysenck, 2009) posited that excessive worry might occupy cognitive resources, leading to insufficient allocation of cognitive resources to tasks and consequently impacting processing efficiency. Shy adolescents, who were more sensitive to potential social threats (LoBue and Pérez-Edgar, 2014), tended to have their attention distracted by social issues unrelated to academic tasks (Alm and Frodi, 2008; Leigh et al., 2021). An excessive worry about interpersonal interactions, social status, or performance in the class took up cognitive resources that should be devoted to the completion of academic tasks. This decreased the efficiency of accomplishing academic tasks, culminating in academic procrastination. Hence, it was evident that shyness is closely associated with academic procrastination in adolescents.

Thirdly, Social Cognitive Theory emphasized the influence of an individual’s subjective expectancy of personal capabilities and the outcome of tasks on behavioral choices (Bandura, 1977). Shy adolescents, characterized by lower self-confidence and self-esteem (Crozier, 1995), may develop pessimistic beliefs, perceiving themselves as destined to fail in academic tasks due to heightened concerns about others’ evaluations and persistent self-doubt (Miller, 1995; Coplan et al., 2004; Geng et al., 2021). This anticipation of failure may lead shy adolescents to psychologically avoid academic tasks, opting to postpone confronting challenges through academic procrastination. In the short term, procrastination may alleviate the pressure of potential failure, providing temporary psychological relief for shy adolescents. However, the trade-off for this comfort was a subsequent compromise in academic performance. In essence, shyness not only impeded adolescents’ academic engagement but also propelled them into a detrimental cycle of procrastination.

Empirical studies have indicated that shy individuals were more prone to experiencing academic challenges, exhibiting lower academic engagement, and achieving suboptimal performance in academic tasks (Hughes and Coplan, 2010; Nikel et al., 2022) – characteristics that align closely with academic procrastination. Based on the above analysis, this study posited that shyness can positively predict academic procrastination among adolescents (Hypothesis 1).

### 1.2 The mediating effect of self-regulation

Self-regulation is a valuable individual resource for adolescents, denoting the conscious adjustment of one’s thoughts, emotions, and actions to achieve goals (Fries et al., 2008). Research indicated that self-regulation is a crucial factor in the learning process and played a pivotal role in achieving academic success (Robson et al., 2020). Individuals with high levels of self-regulation demonstrated unique advantages in learning activities. They can set clear learning goals and efficiently choose appropriate learning strategies. Through continuous assessment of academic progress, they can timely adjust their learning strategies, ensuring success in completing academic tasks (Ziegler and Opdenakker, 2018). Conversely, individuals with low levels of self-regulation exhibited significant disadvantages in the learning process. Their adaptability was notably poor when faced with new changes or challenges in academic tasks. They struggled to adjust states promptly in aspects such as time management, emotional regulation, and distraction elimination. Consequently, they were unable to efficiently accomplish academic goals within limited time, leading to academic procrastination (Park and Sperling, 2012; Valenzuela et al., 2020). Thus, self-regulation negatively predicted academic procrastination.

The relationship between shyness and self-regulation has also garnered scholarly attention. Shy adolescents tended to employ self-protective strategies, such as avoiding social interactions or relying on safety behaviors (Hassan et al., 2021). These strategies may consume significant cognitive and emotional resources, disrupting self-efficacy regarding academic tasks (Nikel et al., 2022). This interference exacerbated difficulties in task planning, time management, emotional regulation, and sustaining attention, thereby reducing individual self-regulation abilities (Leigh et al., 2021).

In conclusion, self-regulation likely served as a mediator in the relationship between shyness and academic procrastination. Adequate and effective self-regulation may contribute to mitigating the adverse impact of shyness on academic procrastination. Previous research has also identified self-regulation as playing a mediating role between personality traits and academic procrastination (Ljubin-Golub et al., 2019). Building on this, this study hypothesized that self-regulation serves as a mediator in the relationship between shyness and academic procrastination (Hypothesis 2).

### 1.3 The moderating effect of self-focused attention

Although shyness influenced adolescent academic procrastination through self-regulation, this impact varies among individuals. Several researchers have emphasized the importance of studying the moderating factors between shyness and social adjustment (Chen et al., 2021; Geng et al., 2021). In this study, we explore potential differences in the relationship between self-focused attention and shyness affecting academic procrastination.

Self-focused attention referred to an individual’s sustained attention and observation of their internal state or external performance, and was considered an important facilitating factor for self-monitoring and self-regulation (Bandura, 2001). Furthermore, according to Self-focused Attention Theory, when individuals focused their attention on themselves, they evaluated their behavior based on relevant standards, goals, and norms (Carver, 2012; Silvia and Eddington, 2012). Subsequently, they adjusted their behavior according to the evaluation results to achieve their goals (van Randenborgh et al., 2010; Silvia et al., 2014). Entering puberty, adolescents experienced an increase in self-awareness. This was accompanied by heightened self-focused attention, directing increased focus towards their thoughts, emotions, physical states, and how they were perceived by others (Steinberg, 2005). In conclusion, self-focused attention may play a facilitating role in adolescents’ self-regulation.

Given that shyness can be perceived as a manifestation of social anxiety to a certain extent (McNeil, 2010), we can derive an understanding of self-focused attention based on our knowledge of anxiety. According to the Self-Focused Cognitive Processes in Models of Social Anxiety/Shyness (Norton and Abbott, 2016), the attentional processes of individuals with social anxiety/shyness interact with self-focused attention, consequently influencing their emotion responses and behavior. Research by Derakshan and Eysenck (2009) further suggests that these individuals tend to allocate cognitive resources predominantly to negative stimuli, potentially impairing their ability to self-regulate. This heightened sensitivity to external evaluations and social threat information, as supported by Geng et al. (2021), LoBue and Pérez-Edgar (2014), Miller (1995), leads individuals with social anxiety/shyness to excessively focus on critiquing their performance in social situations, diverting attention from immediate tasks and developmental needs. Consequently, even with heightened self-focused attention, the facilitating effect on self-regulation may be diminished in this population. Contrastingly, adolescents with low shyness yet heightened self-focused attention, took more recourse to using self-focused attention to meet their developmental requirements, potentially enhancing their self-regulation implementation. That is, the facilitating impact of heightened self-focused attention on self-regulation was more evident. Therefore, self-focused attention played a moderating role in the relationship between shyness and self-regulation, and this moderating effect aligned with the stress-vulnerability hypothesis (Li et al., 2012), whereby higher levels of self-focused attention were more beneficial for low-shy adolescents.

Although researchers have not yet examined the interactive effect of shyness and self-focused attention on predicting adolescent academic development, there is some indirect evidence supporting the stress-vulnerability hypothesis. For instance, researchers have found that self-focused attention elevated effort management in low anxious individuals, thereby enhancing test performance. However, this effect was less pronounced in high anxious individuals (Carver et al., 1983; Eddington and Foxworth, 2012).

Based on theoretical and empirical analyses, self-focused attention may play a moderating role in shyness and self-regulation. Specially, it has a clear enhancement effect for low-shyness adolescents, but the effect is less evident for shy adolescents (Hypothesis 3).

**Figure 1:**
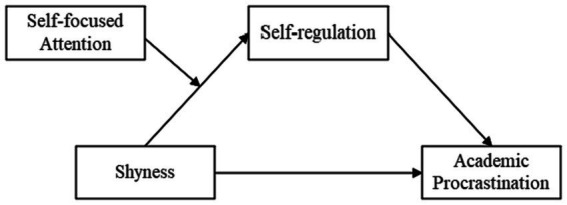
Conceptual model.

The research framework of this study is as follows (see Figure 1).

## 2 Materials and methods

### 2.1 Participants

The study participants were chosen through cluster sampling, with five regular middle schools randomly selected in Shandong Province, China. Two classes from each of grades one and two, and one class from grade three in each school, were randomly chosen for the survey. Data was collected within classrooms, with 1,431 questionnaires distributed and 1,279 valid responses obtained, yielding an 89.38% effective response rate. The gender distribution included 632 males (49.41%) and 647 females (50.59%). Among the participants, 544 were only children (42.5%) and 735 were not (57.5%). In terms of residence, 781 participants lived in rural areas (61.1%) and 498 resided in urban areas (38.9%). Grade-wise distribution included 543 participants in grade one (42.5%), 559 in grade two (43.7%), and 177 in grade three (13.8%). The mean age of these adolescents was 13.49 ± 1.002. The study was approved by the Ethics Committee of Taishan University and informed consent was obtained from students, parents, and teachers after explaining the study’s nature, objectives, potential benefits, and possible risks.

### 2.2 Measures

#### 2.2.1 Shyness

We used the Revised Cheek and Buss Shyness Scale (RCBS; Cheek, 1983) to assess adolescent shyness, which has been widely used in Chinese students (Chen et al., 2021; Geng et al., 2021). Responses were recorded on a 5-point Likert scale across 13 items, resulting in a total score range of 13–65. Higher scores mean higher level of shyness. Sample items include “I am quite poor in social situations.” In this study, Cronbach’s α for shyness was 0.90. The translation process, employing a translation-back translation approach, addressed inconsistencies, ensuring linguistic uniformity. Two developmental psychology professors evaluated the language expressions and confirmed their appropriateness.

#### 2.2.2 Self-focused attention

The Self-focused Attention Scale (SFAS), developed by Kiropoulos and Klimidis (2006), and revised by Xiao (2010), was used in this study. The questionnaire uses a 5-point Likert scale across 17 items, with scores ranging from 17 to 85. Higher scores represent higher level of self-focused attention. Sample items include “I care a lot about the way I present myself physically.” This scale has shown good reliability and validity in previous research (Ding et al., 2021). In this study, Cronbach’s *α* for self-focused attention was 0.75.

#### 2.2.3 Self-regulation

Adolescents’ self-regulation was assessed by Self-regulation Questionnaire (SRQ; Gao, 2011). The questionnaire encompasses three factors: motivation (e.g., I set goals before the beginning of each week.), strategy (e.g., For personal matters, I often do not set specific deadlines for completion.), and behavior (e.g., I often argue with classmates), and consists of 38 items. The questionnaire employs a 4-point Likert scale, with scores ranging from 38 to 190, where higher scores indicate greater self-regulation ability. The questionnaire has demonstrated good reliability and validity in previous research (Yu et al., 2019). In this study, Cronbach’s *α* for self-regulation was 0.92.

#### 2.2.4 Academic procrastination

We used the Academic Procrastination Inventory (API), developed by Aitken (1982) and adapted by Chen et al. (2008), to assess students’ academic procrastination behavior. The questionnaire uses a 5-point Likert scale across 13 items, with higher scores indicating more severe procrastination. Sample items include “I always wait until the study tasks can no longer be postponed before starting them.” Cronbach’s *α* for academic procrastination was 0.80. The API was a suitable tool for assessing academic procrastination and demonstrated good reliability and validity among Chinese students (Chen et al., 2008; Zheng and Xu, 2022).

### 2.3 Statistical analysis

All data analyses were carried out using SPSS 23.0 and the SPSS macro program PROCESS (Hayes, 2013). Initially, Harman’s single-factor test was applied to assess common-method bias associated with self-report questionnaires. Following this, descriptive statistics and correlation analyses were conducted to explore the associations between variables. Subsequently, the SPSS macro program PROCESS (Model 4) was employed to evaluate the mediation effect of self-regulation. Lastly, conditional process analysis was performed using the SPSS macro program PROCESS (Model 7) to confirm whether self-focused attention moderated the mediation model.

## 3 Results

### 3.1 Common method biases test

We employed Harman’s single-factor test to assess for the potential impact of common method bias. The results of this analysis revealed 21 factors with characteristic roots exceeding one, and the variance explained by the first factor amounted to 16.98%, falling below the conventional threshold of 40%. This demonstrated there was no pronounced methodological bias in this study.

### 3.2 Correlation analysis among study variables

Table 1 lists the means, standard deviations, and correlation matrix for the variables. All variables exhibited statistically significant correlations in conceptually expected ways. Shyness was positively associated with academic procrastination and negatively associated with self-regulation. Self-regulation was negatively associated with academic procrastination. Self-focused attention was positively associated with self-regulation.

**Table 1:** Descriptive statistics and Pearson correlations among study variables (*N* = 1,279).

	Variables	M	SD	1	2	3	4	5	6	7	8
1	Gender	1.510	0.500	1.000							
2	Shyness	34.459	8.324	−0.047	1.000						
3	SFA	57.388	9.709	0.045	0.021	1.000					
4	Motivational SR	35.455	6.651	0.121^**^	−0.218^**^	0.107^**^	1.000				
5	Behavioral SR	35.208	4.797	0.196^**^	−0.186^**^	0.016	0.620^**^	1.000			
6	Strategic SR	36.518	5.968	0.153^**^	−0.230^**^	0.177^**^	0.747^**^	0.600^**^	1.000		
7	Total SR	95.713	13.847	0.171^**^	−0.244^**^	0.119^**^	0.904^**^	0.808^**^	0.900^**^	1.000	
8	AP	31.004	8.925	−0.147^**^	0.232^**^	−0.102^**^	−0.634^**^	−0.611^**^	−0.620^**^	−0.702^**^	1.000

^*^p < 0.05 (two-tailed) and ^**^p < 0.01(two-tailed). Gender was coded: 1 = boy and 2 = girl. SFA, self-focused attention; SR, self-regulation; AP, academic procrastination. Motivational SR, behavioral SR, and strategic SR are the three factors of SR.

### 3.3 Moderated mediation effect analysis

Using PROCESS and employing bias-corrected non-parametric percentile Bootstrap method for estimating confidence intervals of coefficients, we investigated the association between shyness and academic procrastination while controlling for the influence of gender.

Firstly, we used the PROCESS macro (Model 4) to examine the mediating role of self-regulation in the relationship between shyness and academic procrastination. The results indicated that shyness was negatively associated with on self-regulation (*β* = −0.236, SE = 0.027, *p* < 0.01) and self-regulation was negatively associated with academic procrastination (*β* = −0.681, SE = 0.021, *p* < 0.01). Moreover, shyness had a significant effect on academic procrastination through self-regulation (*β* = 0.161, SE = 0.021, *p* < 0.01). Shyness also had a direct significant effect on academic procrastination (*β* = 0.225, SE = 0.027, *p* < 0.01). Consequently, we concluded that self-regulation partially mediated the relationship between shyness and academic procrastination, with the mediation effect accounting for 71.429% of the total effect (see Table 2).

**Table 2:** Mediation analysis (*N* = 1,279).

Dependent variables	Independent variables	*β*	SE	*t*	95% CI	*R* ^2^	*F*
AP	Gender	−0.272	0.054	−5.036^**^	[−0.378, −0.166]	0.072	49.538^**^
	Shyness	0.225	0.027	8.339^**^	[0.172, 0.278]		
SR	Gender	0.319	0.054	5.946^**^	[0.214, 0.424]	0.085	59.168^**^
	Shyness	−0.236	0.027	−8.820^**^	[−0.289, −0.184]		
AP	Gender	−0.055	0.040	−1.356	[−1.134, 0.024]	0.497	420.046^**^
	Shyness	0.064	0.020	3.125^**^	[0.024, 0.104]		
	SR	−0.681	0.021	−32.825^**^	[−0.722, −0.641]		

^*^p < 0.05 (two-tailed) and ^**^p < 0.01 (two-tailed). Bootstrap sample size = 5,000. Gender was coded: 1 = boy and 2 = girl. SFA, self-focused attention; SR, self-regulation; AP, academic procrastinations; LL, low limit; CI, confidence interval; UL, upper limit.

Subsequently, we employed Model 7 of the SPSS PROCESS macro program to scrutinize the moderated mediation model (see Table 3). Upon the inclusion of self-focused attention in the model, self-focused attention was found to positively predict self-regulation (*β* = 0.112, *t* = 4.213, *p* < 0.01). Additionally, it moderated the first half of the indirect effect (*β* = −0.052, *t* = −2.037, *p* < 0.05).

**Table 3:** Conditional process analysis (*N* = 1,279).

	*β*	SE	*t*	95% CI
Mediator variable model for predicting SR				
Gender	0.306	0.053	5.749^**^	[0.202, 0.410]
Shyness	−0.231	0.027	−8.597^**^	[−0.284, −0.178]
SFA	0.112	0.027	4.213^**^	[0.060, 0.165]
Shyness × SFA	−0.052	0.025	−2.037^*^	[−0.102, −0.002]
Dependent variable model for predicting AP				
Gender	−0.055	0.040	−1.356	[−0.134, 0.024]
Shyness	0.064	0.020	3.125^**^	[0.024, 0.104]
SR	−0.681	0.021	−32.825^**^	[−0.722, −0.641]
Conditional effect	Effect	Boot SE	Boot LLCI	Boot ULCI
M − 1SD	−0.179	0.040	−0.257	−0.101
M	−0.231	0.027	−0.284	−0.178
M + 1SD	−0.283	0.034	−0.350	−0.216

^*^p < 0.05 (two-tailed) and ^**^p < 0.01 (two-tailed). Bootstrap sample size = 5,000. Gender was coded: 1 = boy and 2 = girl. SFA, self-focused attention; SR, self-regulation; AP, academic procrastination; LL, low limit; CI, confidence interval; UL, upper limit.

**Figure 2:**
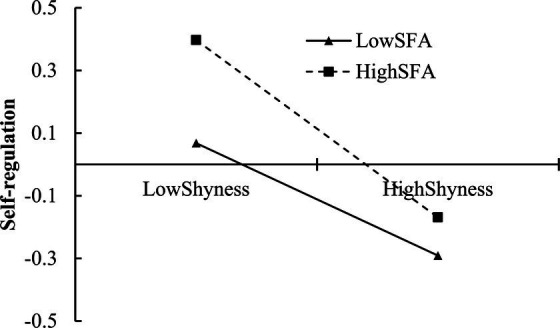
Self-focused attention moderated the relationship between shyness and self-regulation. SFA, self-focused attention.

To clarify the characteristics of the interaction terms, a simple slope test was conducted to analyze the moderating effect of self-focused attention (see Figure 2). Generally, when self-focused attention was low (M − 1SD), a significant negative predictive effect of shyness on self-regulation was evident (*β* = −0.179, *t* = −4.521, *p* < 0.01). Conversely, when self-focused attention was high (M + 1SD), the negative predictive effect of shyness on self-regulation was more pronounced (*β* = −0.283, *t* = −8.275, *p* < 0.01). This indicated that the predictive effect of shyness on self-regulation varies at different levels of self-focused attention, with higher self-focused attention strengthening the predictive relationship between the two.

## 4 Discussion

This study constructed a moderated mediation model based on Attentional Control Theory and Self-focused Attention Theory, comprehensively investigated the association and potential impact of shyness on academic procrastination in adolescence. The findings revealed that self-regulation partially mediated the relationship between shyness and academic procrastination, and self-focused attention moderated the relationship between shyness and self-regulation.

The findings contributed to a comprehensive understanding of the internal mechanisms of academic procrastination. They not only addressed the knowledge gap regarding the relationship between shyness and academic procrastination but also provided a new perspective on understanding the academic challenges faced by shy adolescents. Moreover, these findings further supported and enriched the viewpoint that personality traits play a significant role in the learning process (De Raad and Schouwenburg, 1996). This study provided valuable insights into methodologies for educational practices and psychological health support.

### 4.1 Mediating effect of self-regulation

From the perspective of differential psychology, procrastination was considered a personality trait (Klingsieck, 2013). The results of this study supported this viewpoint: shyness positively predicted academic procrastination in adolescents, and this positive association can be explained by self-regulation. Shyness diminished individuals’ self-regulation, consequently contributing to the occurrence of academic procrastination. This finding deepened our understanding of the internal mechanisms through which social pressure and emotional distress impact academic achievement. Two points can be considered to explain this result:

On the one hand, shyness negatively predicted adolescent self-regulation, with higher levels of shyness associated with poorer self-regulation. From a cognitive perspective, shy adolescents exhibited lower self-efficacy (Nikel et al., 2022), making it challenging to clarify academic goals and formulate effective plans. On the emotional front, shy adolescents experienced more negative emotions, posing greater challenges in emotional management and making it difficult to regulate emotions for concentration. Regarding social interaction, shy adolescents faced difficulties in academic social interactions, reducing opportunities for external support and collaboration (Chen et al., 2021). Thus, the typical cognitive, emotional, and social interaction patterns in children can shape their abilities to manage emotions, behaviors, and thoughts in the process of goal achievement and environmental adaptation. Personality traits play a crucial role in self-regulation abilities.

On the other hand, self-regulation negatively predicted academic procrastination, aligning with findings in the procrastination research field (Park and Sperling, 2012; Valenzuela et al., 2020). When adolescents can effectively monitor their own behavior and engage in self-assessment, they demonstrate advantages in task planning, time management, and emotional regulation, enabling them to complete academic tasks on time. Conversely, self-regulation failure, accompanied by a malfunction in emotional and psychological resource allocation, leads to academic procrastination. In summary, self-regulation played a crucial bridging role between shyness and academic procrastination.

Furthermore, in recent years, the phenomenon of procrastination has extended beyond the academic domain, sparking widespread interest in various fields, including financial risk and hindrances in career planning (Nguyen et al., 2013). Future research should explore the pivotal roles of other personality factors and self-regulation in procrastination behaviors across different domains, such as finance, medicine, and management. This mediating model offers valuable insights for educators and parents, suggesting that providing education, training, and practical activities focused on enhancing self-regulation skills for shy adolescents can improve their self-regulation, enhance task execution efficiency, effectively reduce the risk of academic procrastination, and better adapt to academic environments.

### 4.2 Moderating effect of self-focused attention

Theoretical and empirical studies indicated that self-focused attention has a significant impact on self-regulation (Carver, 2012; Silvia and Eddington, 2012; Silvia et al., 2014). This study validated this conclusion, demonstrating that self-focused attention facilitates self-regulation. However, previous research on self-focused attention had predominantly focused on areas such as psychotherapy, medicine, and the self. The present study contributed to this exploration by introducing an investigation of self-focused attention and its crucial role in academic development.

Moreover, self-focused attention played a moderating role in the first half of the mediating process of “shyness → self-regulation → academic procrastination.” Specifically, adolescents with high levels of self-focused attention exhibited a heightened negative predictive impact of shyness on self-regulation compared to those with lower levels. This implies that heightened self-focused attention attenuated the self-regulation of highly shy adolescents, consequently exacerbating academic procrastination. Consequently, it is imperative to approach the dual role of self-focused attention in this mediation process cautiously: while it serves as a protective factor by bolstering self-regulation and mitigating academic procrastination, excessive self-focused attention in the presence of heightened shyness may exacerbate this risk, resulting in diminished self-regulation and heightened likelihood of academic procrastination. Hence, it is overly simplistic to assume a universally beneficial role for self-focused attention. Instead, its efficacy appears to be more pronounced among less shy adolescents, whereas its protective effects are constrained for those with heightened shyness. This observation aligns seamlessly with the stress-vulnerability hypothesis, suggesting that the resilience of protective factors (e.g., self-focused attention) may wane as risks (e.g., shyness) escalate to a certain threshold (Li et al., 2012). Consequently, optimism regarding the beneficial effects of self-focused attention as a protective factor should be tempered, while the detrimental effects of shyness as a risk factor warrant heightened scrutiny. In summary, this discovery underscored the critical importance of comprehending adolescent academic procrastination at the level of individual differences.

In educational practice, educators should foster adolescents’ self-focused attention to enhance their self-regulation and academic performance. This entails instructing them on effectively utilizing self-focused attention to monitor and adjust their cognition, emotions, and behaviors, thereby better adapting to various situational and task demands. Furthermore, for shy adolescents, educators need to pay special attention to their emotional management and provide psychological support to alleviate anxiety and promote self-regulation, thereby reducing academic procrastination. Most importantly, the findings of this study highlight the interconnected nature of various internal factors influencing adolescents’ academic procrastination, rather than acting independently. Therefore, future interventions should not solely focus on one aspect. Instead, integrated and systematic interventions are preferable, targeting three key areas: shyness, self-focused attention, and self-regulation. This comprehensive approach aims to achieve optimal intervention outcomes and promote the healthy development of adolescents.

### 4.3 Limitations and future directions

This study encountered several limitations. Firstly, due to its cross-sectional nature, causal relationships between variables could not be established. Future research should employ longitudinal designs to explore bidirectional and causal relationships among variables. Secondly, the reliance solely on self-reported data may introduce bias. Subsequent studies should incorporate data from diverse sources, including parents, teachers, and peers, to achieve a more comprehensive and objective measurement through cross-validation of information. Furthermore, future studies should endeavor to explore innovative measurement approaches and experimental designs to enhance the understanding of academic procrastination and its associated impact variables, particularly focusing on self-regulation. The integration of neuroimaging techniques such as functional magnetic resonance imaging (fMRI) or electroencephalography (EEG) holds promise for revealing neural correlates of self-regulation processes (Thibault et al., 2015). Additionally, cognitive training interventions or virtual reality simulations may provide valuable insights into the flexibility of self-regulation and inform targeted interventions (Buckley et al., 2014). By incorporating state-of-the-art methodologies, researchers can deepen their understanding of the mechanisms underlying academic procrastination, thus advancing more effective interventions and applications.

## Data availability statement

The original contributions presented in the study are included in the article/Supplementary material, further inquiries can be directed to the corresponding author.

## Ethics statement

The studies involving humans were approved by Ethics Committee of Taishan University. The studies were conducted in accordance with the local legislation and institutional requirements. Written informed consent for participation in this study was provided by the participants’ legal guardians/next of kin.

## Author contributions

HS: Conceptualization, Writing – original draft, Writing – review & editing, Data curation, Funding acquisition, Methodology. YY: Conceptualization, Writing – original draft, Writing – review & editing, Project administration, Resources, Supervision. CP: Supervision, Writing – review & editing.
